# Supplementary material for: Corticotropin-releasing hormone modulates NREM sleep consolidation through the thalamic reticular nucleus
Source: Nat Commun. 2025 Aug 19;16:7720. doi: 10.1038/s41467-025-63118-6 (PMC12365041; doi:10.1038/s41467-025-63118-6)
Supplement: Supplementary file 1 — Supplementary Information [file 41467_2025_63118_MOESM1_ESM.pdf]

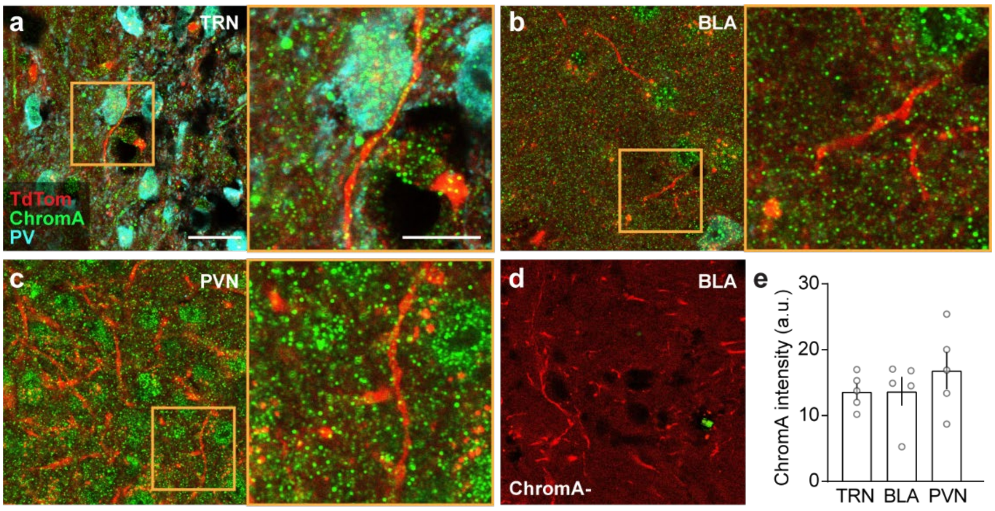

Supplementary Fig. 1. Large dense core vesicles are present in CRH-releasing fibers in the TRN

a-c Example confocal images showing immunoreactivity for Chromogranin A (ChromA), TdTomato (TdTom) and parvalbumin (PV), marking large dense core vesicles, CRH-releasing fibers and PV-positive cells, respectively, in TRN, BLA and PVN from a CRH-IRES-Cre X Ai27D mouse. Low magnification images (scale bar = 20 µm) are shown with higher magnification images (scale bar = 10 µm) of the marked area. Intensity of individual pseudo-coloured channels were adjusted before merging for visualization purpose.

d Negative control immunostaining for ChromA.

e Quantification of ChromA immunoreactivity in TdTom-positive fibers in the three brain regions. Statistical analysis was performed by Friedman test. For additional statistical information, see Supplementary Table 1. Source data are provided as a Source Data file.

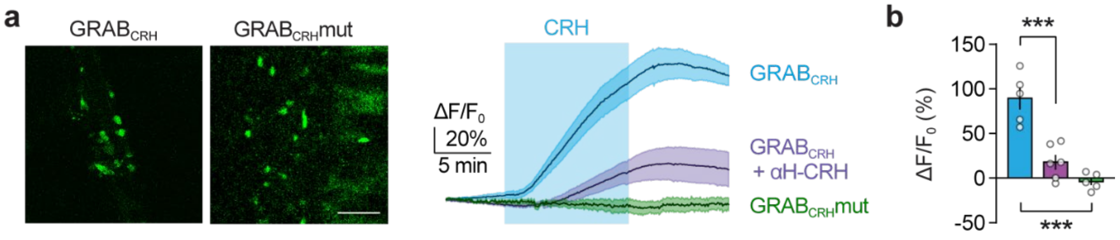

Supplementary Fig. 2. *Ex-vivo* validation of the $GRAB_{CRH}$ sensor

a Left, example images of basal fluorescence in TRN cells infected with $GRAB_{CRH}$ and $GRAB_{CRH}$mut (scale bar = 100 µm). Right, average responses of TRN neurons to bath-applied CRH (100 nM for $GRAB_{CRH}$ and 500 nM for $GRAB_{CRH}$mut), expressed as % change in fluorescence compared to baseline ($\Delta F/F_0$). An additional experimental series was included in which $GRAB_{CRH}$-infected slices were preincubated with the CRHR blocker αH-CRH (100 nM).

b Quantification of max $\Delta F/F_0$ in the three groups, validating $GRAB_{CRH}$ as a reporter of CRH.

Data are represented as mean ± SEM. All scale bars are 100 µm. Statistical analysis was performed by one-way ANOVA with Holm-Šídák's multiple comparisons test, with ***$p < 0.001$. For additional statistical information, see Supplementary Table 1. Source data are provided as a Source Data file.

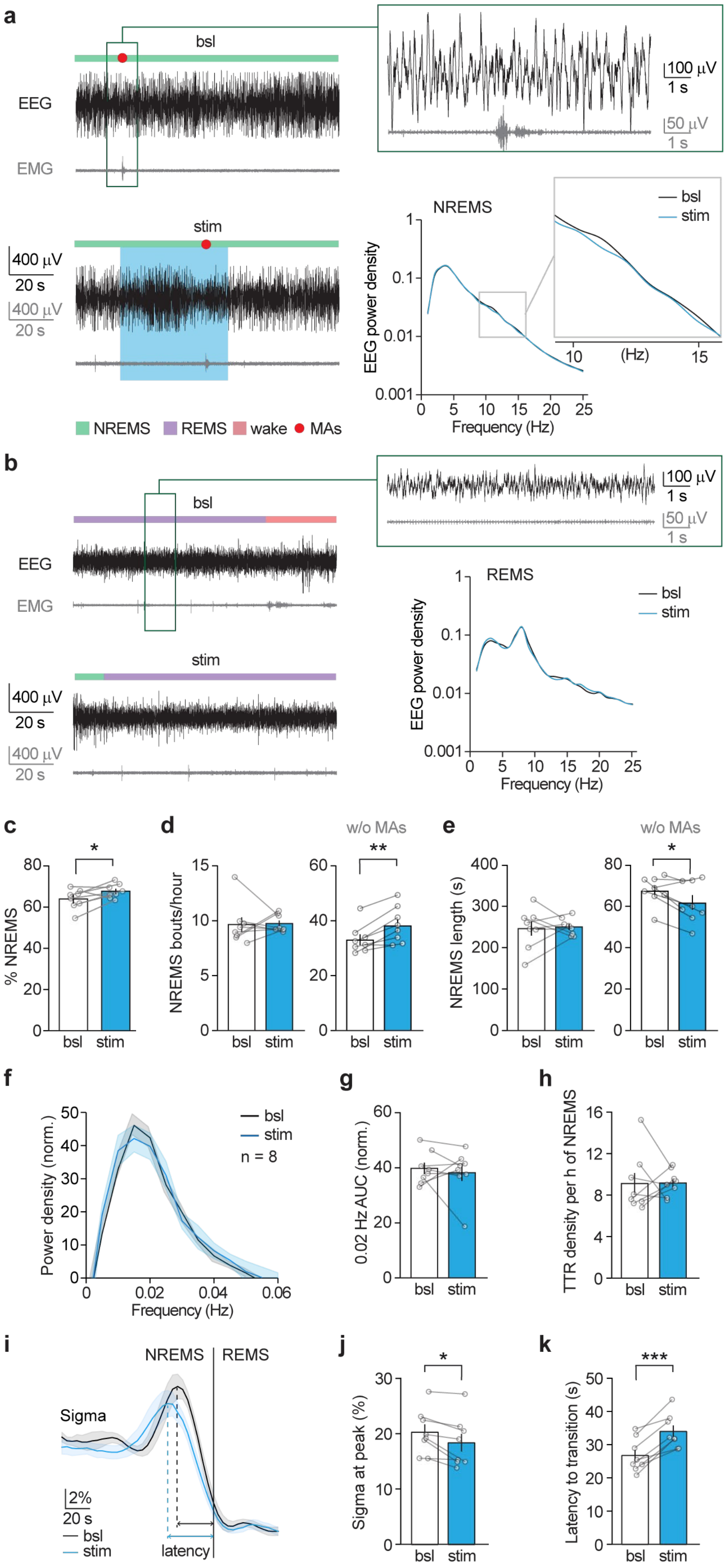
a
bsl
EEG
EMG
100 µV
1 s
50 µV
1 s
stim
400 µV
20 s
400 µV
20 s
NREMS
EEG power density
Frequency (Hz)
bsl
stim
(Hz)
NREMS REMS wake MAs
b
bsl
EEG
EMG
stim
REMS
c
% NREMS
d
NREMS bouts/hour
w/o MAs
e
NREMS length (s)
w/o MAs
bsl stim
f
Power density (norm.)
Frequency (Hz)
n = 8
g
0.02 Hz AUC (norm.)
h
TTR density per h of NREMS
i
NREMS REMS
Sigma
2%
20 s
latency
j
Sigma at peak (%)
k
Latency to transition (s)

Supplementary Fig. 3. Optogenetic stimulation CRH release affects NREMS architecture but not sigma infraslow periodicity

a, **b** Example portions of EEG/EMG traces (with enlarged scales in the insets) and NREMS and REMS power spectra during baseline (bsl) and stimulation (stim) session in a CRH-IRES-Cre X Ai27D mouse.
c Boosting CRH release with photostimulation of Channelrhodopsin (ChR2) increased total time spent in NREMS, without changes in the number of NREMS bouts (**d**), or the average length of NREMS bouts (**e**). However, if MAs are considered as interruptions of NREMS episodes (w/o MAs), significant differences appear, consistent with increased NREMS fragmentation.
f Power spectral density analysis of the contributing frequencies within the sigma power band throughout NREMS shows the expected peak at 0.02 Hz for baseline (black trace) and stimulation with ChR2 (blue trace).
g Area under the curve (AUC) for ± 0.015 Hz around 0.02 Hz, representing the strength of the infraslow oscillation, shows no change between baseline and ChR2 stimulation.
h Photostimulation did not change the number of transitions to REMS per hour of NREMS.
i-k Analysis of NREMS-REMS transitions using a minimum bout length = 2 for REMS episode detection confirms the results found with minimum bout length = 5, i.e., significant reduction in sigma peak and increased latency to transition upon photostimulation.
Data are represented as mean ± SEM, n = 8. Statistical analysis was performed by two-tailed paired t-tests and Wilcoxon test, with $*p < 0.05$, $**p < 0.01$, $***p < 0.001$. For additional statistical information, see Supplementary Table 1. Source data are provided as a Source Data file.

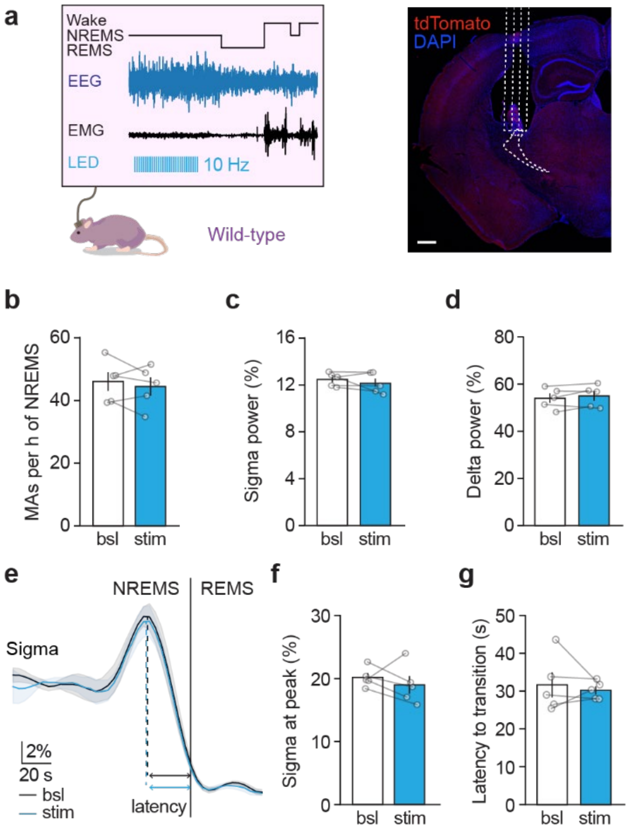

Supplementary Fig. 4. ChR2-WT mice are not affected by the photostimulation protocol

a Wild-type (ChR2-negative) littermates (representative image on the right, scale bar 200 µm) were implanted with optic fibers bilaterally over the TRN and received stimulation with the same protocol as the ChR2-expressing mice (left – 456 nm light stimulation at 10 Hz every 50 s of closed-loop detected NREMS with at least 50 s between stimulations). The stimulation protocol did not change the number of microarousals (MAs) (**b**), sigma power (**c**) or delta power (**d**) throughout NREMS.

e The sigma surge at the transition to REMS showed no change in the peak amplitude (**f**) nor latency to REMS entry (**g**).

Data are represented as mean ± SEM, n = 5. Statistical analysis was performed by two-tailed paired t-tests, with $p > 0.05$. For additional statistical information, see Supplementary Table 1. Source data are provided as a Source Data file.

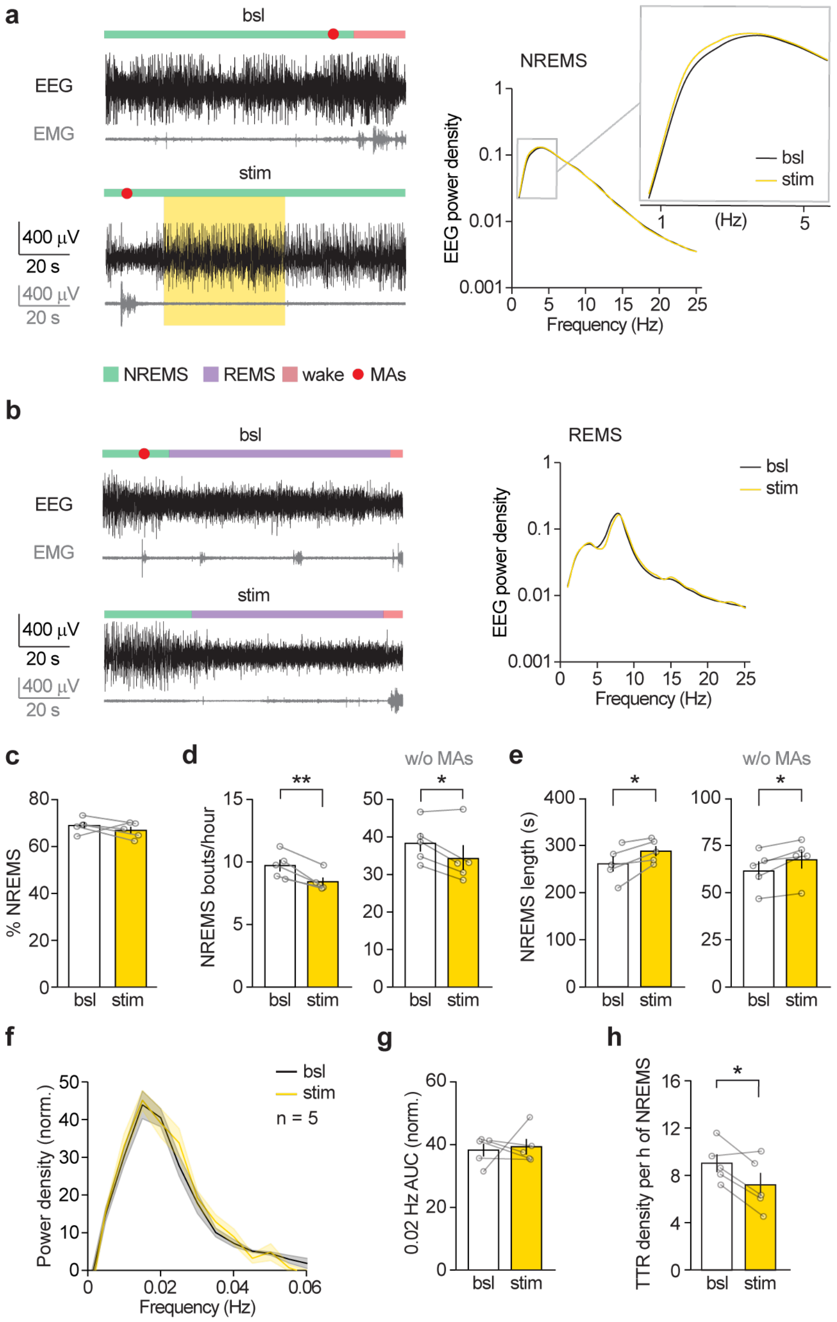

Supplementary Fig. 5. Optogenetic inhibition of CRH release affects NREMS architecture but not sigma infraslow periodicity

a, **b** Example portions of EEG/EMG traces and NREMS and REMS power spectra during baseline (bsl) and stimulation (stim) session in a CRH-IRES-Cre mouse expressing Parapinopsin (PPO) in CHR-releasing neuron targeting TRN.

c Decreasing CRH release with photostimulation of PPO did not have an effect on the total time spent in NREMS. However, (**d**) reducing CRH release in the sensory TRN induced fewer bouts of NREMS (**e**) of longer duration. These differences persisted if MAs were considered as interruption of NREMS episodes.

f, **g** No change was found in the strength of the infraslow oscillation of sigma with PPO photoinhibition of CRH axons.

h Photoinhibition decreased the number of transitions to REMS per hour of NREMS.

Data are represented as mean ± SEM, n = 5. Statistical analysis was performed by two-tailed paired t-tests, with *p < 0.05, **p < 0.01. For additional statistical information, see Supplementary Table 1. Source data are provided as a Source Data file.

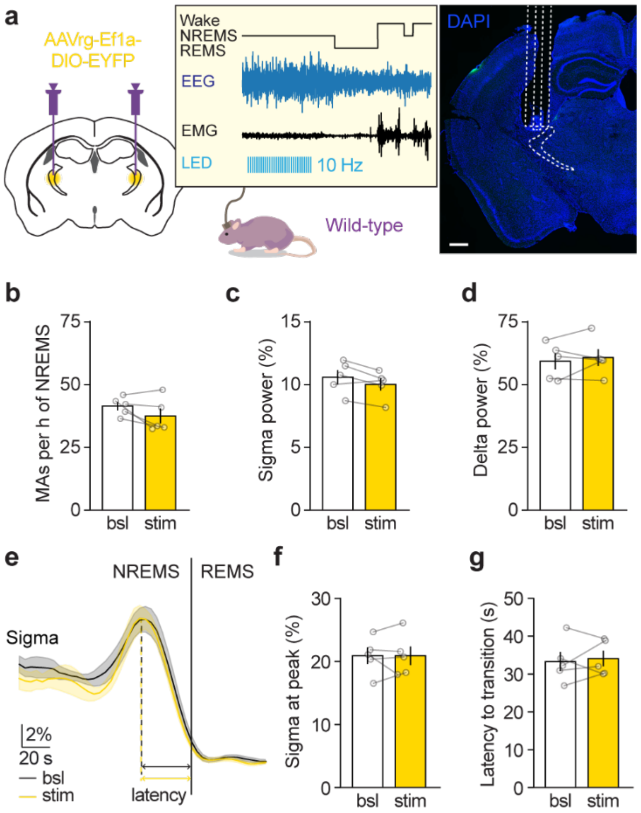

Supplementary Fig. 6. EYFP control mice are not affected by the photoinhibition protocol

a CRH-IRES-Cre mice virally expressing yellow fluorescent protein (EYFP) as a control reporter for Parapinopsin (PPO) in all CRH projections to the TRN were implanted with optic fibers bilaterally over the TRN (right confocal image, scale bar 500 µm) and received stimulation with the same protocol as the PPO-expressing mice (middle – 456nm light stimulation at 10 Hz every 50 s of closed-loop detected NREMS with at least 50 s between stimulations).

b The stimulation protocol did not change the number of microarousals (MAs), sigma power (**c**) or delta power (**d**) throughout NREMS.

e The sigma surge at the transition to REMS also showed no change in the peak amplitude (**f**) or latency to REM sleep (**g**).

Data are represented as mean ± SEM, n = 5. Statistical analysis was performed by two-tailed paired t-tests, with $p > 0.05$. For additional statistical information, see Supplementary Table 1. Source data are provided as a Source Data file.

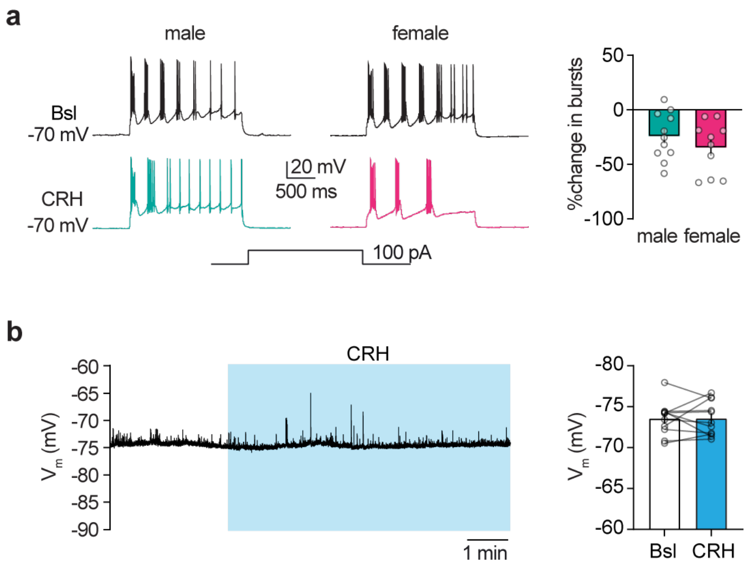

Supplementary Fig. 7. CRH affects TRN bursting in both sexes without altering the membrane potential

a Example voltage traces from TRN neurons in a male and a female mouse before (Bsl) and after (CRH) application of 500 nM CRH and quantification of the %change in the number of bursts, showing no difference between sexes.

b Example recording and quantification of membrane potential (V_m) in TRN neurons prior to (Bsl) and after (CRH) application of 500 nM CRH, indicating lack of CRH effect on V_m.

Data are represented as mean ± SEM. Statistical analysis was performed by two-tailed unpaired (**a**) or paired (**b**) t-tests, with $p > 0.05$. For additional statistical information, see Supplementary Table 1. Source data are provided as a Source Data file.

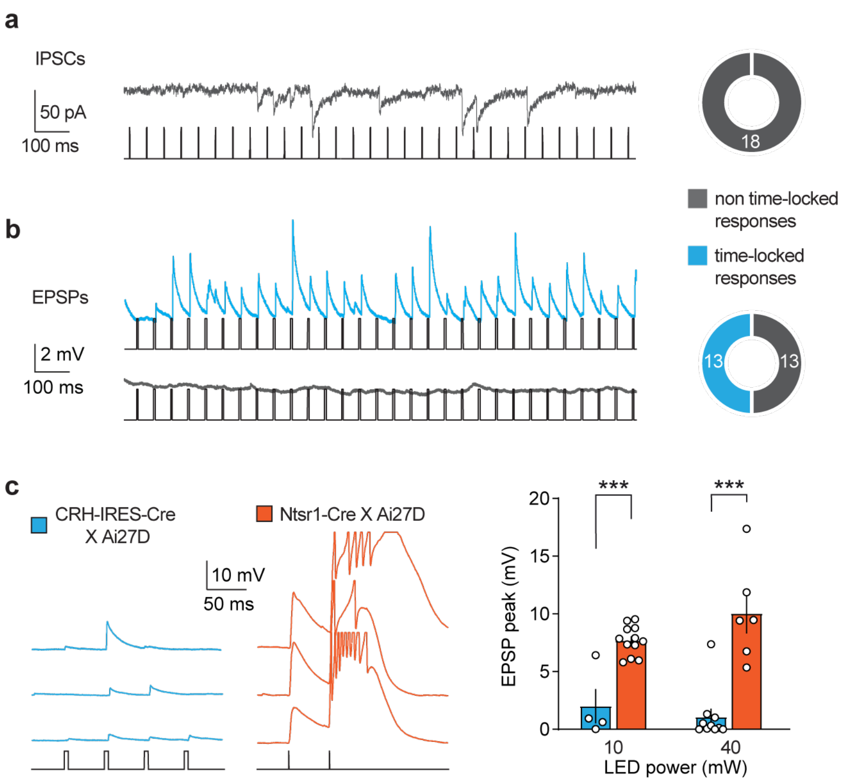

Supplementary Fig. 8. Excitatory but not inhibitory postsynaptic responses are elicited by photoactivation of CRH afferents

a Patch-clamp recordings of inhibitory postsynaptic currents (IPSCs) during photostimulation of CRH afferents (20 Hz train of blue light flashes at 470 nm for 22 s and 1 - 5 ms pulse width) in CRH-IRES-Cre X Ai27D mice. TRN neurons were held at -70 mV in the presence of the AMPAR and NMDAR antagonists DNQX (10 µM) and D,L-APV (50 µM). Photostimulation is indicated by the TTL black trace. No cells exhibited time-locked IPSCs as represented in the gray trace and left pie chart, suggesting that CRH might not be co-released with GABA in TRN.

b Patch-clamp recordings of excitatory postsynaptic potentials (EPSPs) during photostimulation of CRH afferents (20 Hz train of blue light flashes at 470 nm for 22 s and 1 - 5 ms pulse width) in CRH-IRES-Cre X Ai27D mice. TRN neurons were held -70 mV in the presence of the $GABA_AR$ antagonist picrotoxin (0.1 mM). Photostimulation is indicated by the TTL black trace. Two example traces from cells categorized as exhibiting time-locked (blue trace) or non time-locked (gray trace) responses. Cells in which time-locked EPSPs could not be distinguished from spontaneous activity that were present from baseline were included in this category as well, leading to 50% of recordings with time-locked EPSPs.

c Comparison of EPSPs evoked in TRN neurons by photostimulation in CRH-IRES-Cre X Ai27D mice and Ntsr1 X Ai27D mice. Left, examples from three different neurons in each mouse line. Spikes elicited by low-threshold bursting in Ntsr1 X Ai27D mice are clipped. LED flashes had a duration of 1-5 ms in CRH-IRES-Cre X Ai27D mice and 100 µs in Ntsr1 X Ai27D mice. Right, quantification of EPSP peak amplitude using different LED power intensities. For CRH-IRES-Cre X Ai27D mice, only recordings with time-locked responses are included.

Data are represented as mean ± SEM. Statistical analysis was performed by Mann-Whitney test or two-tailed unpaired t-test, with ***$p \leq 0.001$. For additional statistical information, see Supplementary Table 1. Source data are provided as a Source Data file.

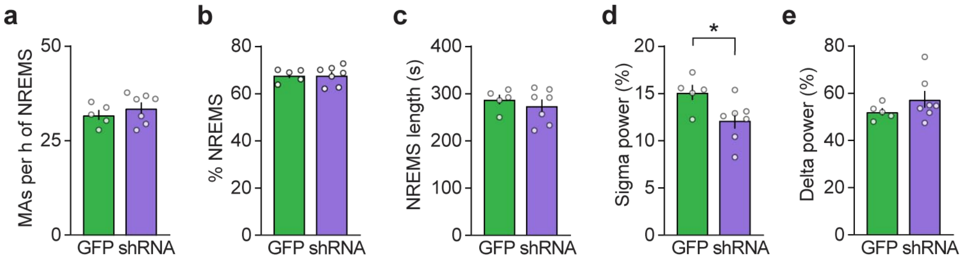

Supplementary Fig. 9. CRHR1 downregulation in TRN does not cause major sleep alterations

a-c CRHR1 downregulation in TRN via shRNAmir virus used to knock-down (KD) CRHR1 did not alter the number of MAs per h of NREMS (MAs, **a**), the % of NREMS (**b**) or the length of NREMS bouts (**c**).

d, **e** CRHR1 downregulation in TRN decreased sigma power (**d**), but did not affect delta power (**e**).

Data are represented as mean ± SEM. Statistical analysis was performed by two-tailed unpaired t-tests, with $p < 0.05$. For additional statistical information, see Supplementary Table 1. Source data are provided as a Source Data file.

Main Figures

Figure	Measure	Subjects	Analysis	Factors	Statistic value	P value	Effect size	Post hoc test	Data normally distributed
1e	CRHR1 mRNA/DAPI	TRN vs CA3 vs BLA (n = 11)	Repeated measures one-way ANOVA		F (1.83, 18.33) = 14.27	ANOVA p = 0.0002	η^2 = 0.5879	Holm-Šídák's multiple comparisons test:<br>TRN vs CA3 p = 0.0037<br>TRN vs BLA p = 0.0037	Yes
1f	CRHR1 mRNA/DAPI	Males (n = 6) vs Females (n = 5)	Unpaired two-tailed t-test		t (9) = 0.2839	p = 0.7829	η^2 = 0.0088		Yes
1i	CRHR1 mRNA/DAPI	PV+ vs SOM+ TRN neurons (n = 11)	Paired two-tailed t-test		t (10) = 8.329	p < 0.0001	η^2 = 0.8740		Yes
3c right	Peak to peak amplitude (z-score)	$GRAB_{CRH}$ vs $GRAB_{CRH}$mut (n = 4)	Paired two-tailed t-test		t (3) = 3.466	p = 0.0405	η^2 = 0.8002		Yes
4c	MAs/hour of NREMS	ChR2 TGs bsl vs stim (n = 8)	Paired two-tailed t-test		t (7) = 5.330	p = 0.0011	η^2 = 0.8023		Yes
4d	Sigma relative power at NREMS	ChR2 TGs bsl vs stim (n = 8)	Paired two-tailed t-test		t (7) =3.258	p = 0.0139	η^2 = 0.6026		Yes
4e	Delta relative power at NREMS	ChR2 TGs bsl vs stim (n = 8)	Paired two-tailed t-test		t (7) = 1.471	p = 0.1848	η^2 = 0.2361		Yes
4g	Sigma peak relative power amplitude at TTR	ChR2 TGs bsl vs stim (n = 8)	Paired two-tailed t-test		t (7) = 2.911	p = 0.0226	η^2 = 0.5476		Yes
4h	Latency to REMs from sigma peak at TTR (s)	ChR2 TGs bsl vs stim (n = 8)	Paired two-tailed t-test		t (7) = 6.172	p = 0.0005	η^2 = 0.8448		Yes
5d	MAs/hour of NREMS	PPO bsl vs stim (n = 5)	Paired two-tailed t-test		t (4) = 3.687	p = 0.0211	η^2 = 0.7726		Yes
5e	Sigma relative power at NREMS	PPO bsl vs stim (n = 5)	Paired two-tailed t-test		t (4) = 0.5269	p = 0.6262	η^2 = 0.06489		Yes
5f	Delta relative power at NREMS	PPO bsl vs stim (n = 5)	Paired two-tailed t-test		t (4) = 3.070	p = 0.0373	η^2 = 0.7021		Yes
5h	Sigma peak relative power amplitude at TTR	PPO bsl vs stim (n = 5)	Paired two-tailed t-test		t (4) = 1.544	p = 0.1976	η^2 = 0.3733		Yes
5i	Latency to REMS from sigma peak at TTR (s)	PPO bsl vs stim (n = 5)	Paired two-tailed t-test		t (4) = 1.523	p = 0.2023	η^2 = 0.3672		Yes
6a right	Nr. of bursts in TRN	Bsl vs CRH 1 µM (n = 9)	Repeated measures two-way ANOVA	Current step x treatment	interaction F (8, 72) = 2.241;<br>current step F (8, 72) = 1.331;<br>treatment F (8, 72) = 34.14	p = 0.0339<br>p = 0.2421<br>p < 0.0001		Holm-Šídák's multiple comparisons test:<br>50 pA p = 0.0087<br>100 pA p < 0.0001	Yes
6b	%change in bursts averaged for current steps 50-125 pA	CRH concentrations: control 0 nM (n = 12), 10 nM (n = 10), 200 nM (n = 11), 500 nM (n = 10), 1 µM (n = 9)	One-way ANOVA		F (4, 47) = 5.946	p = 0.0006	η^2 = 0.3360	Holm-Šídák's multiple comparisons test:<br>0 nM vs 200 nM p = 0.0426<br>0 nM vs 500 nM p = 0.0330<br>0 nM vs 1 µM p < 0.0001	Yes

6c right	%change in bursts	WT (n = 10) vs CRH-ChR2 TG (n = 11)	Repeated measures two-way ANOVA	stimulation x genotype	interaction F (2, 38) = 2.410;<br>stimulation F (1.931, 36.70) = 5.594;<br>genotype F (1, 19) = 4.559	p = 0.1034<br>p = 0.0081<br>p = 0.0460		Holm-Šídák's multiple comparisons test:<br>Bsl vs 5 mins post-stim p = 0.0448<br>Bsl vs 10 mins post-stim p = 0.0052	Yes
6d left	%change in bursts	CRH 500 nM (n = 10) vs CRH 500 nM + NBI35965 3 µM (n = 8)	Two-tailed Mann-Whitney		U (4)	p = 0.0005			No
6d right	%change in bursts	CRH-ChR2 TG (n = 11) vs + CRH-ChR2 TG + NBI35965 3 µM (n = 15)	Two-tailed Mann-Whitney		U (27.50)	p = 0.0028			No
6e	SK2 charge, absolute values	Bsl vs end, Ctr (n = 7)	Paired one-tailed t-test		t (6) = 1.906	p = 0.1053	η^2 = 0.3771		Yes
	SK2 charge, absolute values	Bsl vs end, CRH 500 nM (n = 8)	Paired one-tailed t-test		t (7) = 1.107	p = 0.305	η^2 = 0.149		Yes
	%change in SK2 charge	Ctr (n = 7) vs CRH 500 nM (n = 8)	Repeated measures two-way ANOVA	Main effect of CRH	F (1, 13) = 0.0677	p = 0.7987			Yes
	Ca_V3 charge, absolute values	Bsl vs end, Ctr (n = 7)	Paired one-tailed t-test		t (6) = 0.2734	p = 0.794	η^2 = 0.0123		Yes
	Ca_V3 charge, absolute values	Bsl vs end, CRH 500 nM (n = 8)	Paired one-tailed t-test		t (7) = 2.079	p = 0.0762	η^2 = 0.3818		Yes
	%change in Ca_V3 charge	Ctr (n = 7) vs CRH 500 nM (n = 8)	Repeated measures two-way ANOVA	Main effect of CRH	F (1, 13) = 3.234	p = 0.095			No
6f	Tail current, charge	Bsl vs end, CRH 500 nM (n = 7)	Two-tailed Wilcoxon test		W (7) = 10	p = 0.467			No
	Tail current, decay time	Bsl vs end, CRH 500 nM (n = 7)	Two-tailed Wilcoxon test		W (7) = 6	p = 0.687			No
6g	Ca^{2+} current peak, absolute values	Bsl vs end, CRH 500 (n = 9)	Two-tailed Wilcoxon test		W (9) = 45	p = 0.0039			No
	Ca^{2+} current peak, absolute values	Bsl vs end, CRH+NBI (n = 6)	Paired one-tailed t-test		t (5) = 0.189	p = 0.857	η^2 = 0.007		Yes
	%change in Ca^{2+} current peak	CRH 500 nM (n = 9) vs CRH+NBI (n = 6)	Mixed-effect analyses	Main effect of NBI	F (1, 13) = 14.75	p = 0.002			No
6h	%change in Ca^{2+} current peak	CRH 500 nM (n = 5)	Repeated measures one-way ANOVA	Peak number	F (1.515, 6.061) = 48.0	p = 0.0003		Holm-Šídák's multiple comparisons test:<br>peak1 vs peak2 p = 0.0055<br>peak1 vs peak3 p = 0.0003	Yes
7c	%change in bursts	GFP (n = 6) vs shRNAmir (n = 6) TRN neurons	Unpaired one-tailed t-test		t (10) = 2.798	p = 0.0094	η^2 = 0.4390		Yes
7e	MAs/hour of NREMS	GFP mice (n = 5)	Paired one-tailed t-test		t (4) = 5.023	p = 0.0037	η^2 = 0.8632		Yes
7f	Sigma relative power at NREMS	GFP mice (n = 5)	Paired one-tailed t-test		t (4) = 3.585	p = 0.0115	η^2 = 0.7626		Yes
7g	Latency to REMS from sigma peak at TTR (s)	GFP mice (n = 5)	Paired one-tailed t-test		t (4) = 5.950	p = 0.0020	η^2 = 0.8985		Yes
7i	MAs/hour of NREMS	shRNAmir mice (n = 6)	Paired one-tailed t-test		t (5) = 1.243	p = 0.1346	η^2 = 0.2359		Yes

7j	Sigma relative power at NREMS	shRNAmir mice (n = 6)	Paired one-tailed t-test		t (5) = 0.949	p = 0.1931	$\eta^2 = 0.1527$		Yes
7k	Latency to REMS from sigma peak at TTR (s)	shRNAmir mice (n = 6)	Paired one-tailed t-test		t (5) = 0.789	p = 0.2329	$\eta^2 = 0.1108$		Yes

Supplementary Figures

**Figure**	**Measure**	**Subjects**	**Analysis**	**Factors**	**Statistic value**	**P value**	**Effect size**	**Post hoc test**	**Data normally distributed**
1e	ChromA intensity (u.a.)	TRN vs BLA vs PVN (n = 5)	Friedman test		F = 0.4	p = 0.9537			No
2b	CRH-induced increase in $\Delta F/F_0$ (%)	$GRAB_{CRH}$ (n = 5) vs $GRAB_{CRH}$ + αH-CRH (n = 6) vs $GRAB_{CRH}$mut (n = 5)	One-way ANOVA		F (2, 13) = 28.93	p < 0.0001	$\eta^2 = 0.3360$	Holm-Šídák's multiple comparisons test:<br>$GRAB_{CRH}$ vs $GRAB_{CRH}$ + αH-CRH p = 0.0001<br>$GRAB_{CRH}$ vs $GRAB_{CRH}$mut p < 0.0001<br>$GRAB_{CRH}$ + αH-CRH vs $GRAB_{CRH}$mut p = 0.102	Yes
3c	% NREMS	ChR2 TG mice (n = 8)	Paired two-tailed t-test		t (7) = 2.705	p = 0.0304	$\eta^2 = 0.5110$		Yes
3d	NREMS bouts/h<br>NREMS bouts/h (w/o MAs)	ChR2 TG mice (n = 8)	Paired two-tailed t-test		t (7) = 0.0286<br>t (7) = 4.544	p = 0.9780<br>p = 0.0027	$\eta^2 = 0.0001$<br>$\eta^2 = 0.7469$		Yes<br>Yes
3e	NREMS length (s)<br>NREMS length (s) (w/o MAs)	ChR2 TG mice (n = 8)	Paired two-tailed t-test		t (7) = 0.4052<br>t (7) = 2.650	p = 0.6975<br>p = 0.0329	$\eta^2 = 0.0229$<br>$\eta^2 = 0.5008$		Yes<br>Yes
3g	0.02 Hz AUC (norm.)	ChR2 TG mice (n = 8)	Wilcoxon test		W (8) = -8	p = 0.6406			No
3h	TTR density per h of NREMS	ChR2 TG mice (n = 8)	Wilcoxon test		W (8) = 0	p > 0.9999			No
3j	Sigma at peak (%)	ChR2 TG mice (n = 8)	Paired two-tailed t-test		t (7) = 3.473	p = 0.0104	$\eta^2 = 0.6328$		Yes
3k	Latency to transition (s)	ChR2 TG mice (n = 8)	Paired two-tailed t-test		t (7) = 6.594	p = 0.0003	$\eta^2 = 0.8613$		Yes
4b	MAs / hour of NREMS	ChR2 WT mice (n = 5)	Paired two-tailed t-test		t (4) = 0.8169	p = 0.4598	$\eta^2 = 0.1430$		Yes
4c	Sigma relative power at NREMS	ChR2 WT mice (n = 5)	Paired two-tailed t-test		t (4) = 0.9750	p = 0.3848	$\eta^2 = 0.1920$		Yes
4d	Delta relative power at NREMS	ChR2 WT mice (n = 5)	Paired two-tailed t-test		t (4) = 1.174	p = 0.3055	$\eta^2 = 0.2563$		Yes
4f	Sigma peak relative power amplitude at TTR	ChR2 WT mice (n = 5)	Paired two-tailed t-test		t (4) = 0.8551	p = 0.4407	$\eta^2 = 0.1545$		Yes
4g	Latency to REMs from sigma peak at TTR (s)	ChR2 WT mice (n = 5)	Paired two-tailed t-test		t (4) = 0.5106	p = 0.6365	$\eta^2 = 0.0611$		Yes
5c	% NREMS	PPO mice (n = 5)	Paired two-tailed t-test		t (4) = 0.9635	p = 0.3899	$\eta^2 = 0.1884$		Yes
5d	NREMS bouts/h<br>NREMS bouts/h (w/o MAs)	PPO mice (n = 5)	Paired two-tailed t-test		t (4) = 5.942<br>t (4) = 3.109	p = 0.0040<br>p = 0.0359	$\eta^2 = 0.8982$<br>$\eta^2 = 0.7073$		Yes<br>Yes
5e	NREMS length (s)<br>NREMS length (s) (w/o MAs)	PPO mice (n = 5)	Paired two-tailed t-test		t (4) = 3.441<br>t (4) = 3.502	p = 0.0263<br>p = 0.0248	$\eta^2 = 0.7475$<br>$\eta^2 = 0.7541$		Yes<br>Yes
5g	0.02 Hz AUC (norm.)	PPO mice (n = 5)	Paired two-tailed t-test		t (4) = 0.2654	p = 0.8038	$\eta^2 = 0.0173$		Yes

5h	TTR density per h of NREMS	PPO mice (n = 5)	Paired two-tailed t-test		t (4) = 3.197	p = 0.0330	$\eta^2 = 0.7187$		Yes
6b	MAs / hour of NREMS	EYFP mice (n = 5)	Paired two-tailed t-test		t (4) = 1.751	p = 0.1549	$\eta^2 = 0.4338$		Yes
6c	Sigma relative power at NREMS	EYFP mice (n = 5)	Paired two-tailed t-test		t (4) = 2.400	p = 0.0744	$\eta^2 = 0.5901$		Yes
6d	Delta relative power at NREMS	EYFP mice (n = 5)	Paired two-tailed t-test		t (4) = 0.6769	p = 0.5356	$\eta^2 = 0.1028$		Yes
6f	Sigma peak relative power amplitude at TTR	EYFP mice (n = 5)	Paired two-tailed t-test		t (4) = 0.0109	p = 0.9918	$\eta^2 = 0.00002$		Yes
6g	Latency to REMs from sigma peak at TTR (s)	EYFP mice (n = 5)	Paired two-tailed t-test		t (4) = 0.4059	p = 0.7056	$\eta^2 = 0.03956$		Yes
7a	%change in bursts averaged for current steps 50-125 pA	Males (n = 11) vs Females (n = 10)	Unpaired two-tailed t-test		t (18.32) = 1.046	p = 0.309	$\eta^2 = 0.0564$		Yes
7b	Effect of CRH on Vm	Bsl vs end, CRH 500 nM (n = 11)	Paired two-tailed t-test		t (11) = 0.0172	p = 0.987	$\eta^2 = 0.4914$		Yes
8c	EPSP peak (mV)	CRH-IRES-Cre (n = 10) vs Ntsr1-Cre (n = 6)	Two-tailed Mann-Whitney		U (2)	p = 0.001			No
		CRH-IRES-Cre (n = 4) vs Ntsr1-Cre (n = 12)	Unpaired two-tailed t-test		t (14) = 5.53	p < 0.0001	$\eta^2 = 0.6859$		Yes
9a	MAs per h of NREMS	GFP (n = 5) vs shRNA (n = 7)	Unpaired two-tailed t-test		t (10) = 0.865	p = 0.412	$\eta^2 = 0.06835$		Yes
9b	% NREMS	GFP (n = 5) vs shRNA (n = 7)	Unpaired two-tailed t-test		t (10) = 0.001	p = 0.999	$\eta^2 = 3.391e\text{-}008$		Yes
9c	NREMS length (s)	GFP (n = 5) vs shRNA (n = 7)	Unpaired two-tailed t-test		t (10) = 0.728	p = 0.483	$\eta^2 = 0.05028$		Yes
9d	Sigma power (%)	GFP (n = 5) vs shRNA (n = 7)	Unpaired two-tailed t-test		t (10) = 2.445	p = 0.0346	$\eta^2 = 0.3741$		Yes
9e	Delta power (%)	GFP (n = 5) vs shRNA (n = 7)	Unpaired two-tailed t-test		t (10) = 1.189	p = 0.262	$\eta^2 = 0.1238$		Yes

Supplementary Table 1. Details on statistical analyses
