# Supplementary material for: Corticotropin-releasing hormone modulates NREM sleep consolidation through the thalamic reticular nucleus
Source: Nat Commun. 2025 Aug 19;16:7720. doi: 10.1038/s41467-025-63118-6 (PMC12365041; doi:10.1038/s41467-025-63118-6)
Supplement: Supplementary file 2 — Description of Additional Supplementary Files [file 41467_2025_63118_MOESM2_ESM.pdf]

Description of Additional Supplementary Files

Supplementary Data 1: contains sequence of the shRNA used in the study.
