# Supplementary material for: Corticotropin-releasing hormone modulates NREM sleep consolidation through the thalamic reticular nucleus
Source: Nat Commun. 2025 Aug 19;16:7720. doi: 10.1038/s41467-025-63118-6 (PMC12365041; doi:10.1038/s41467-025-63118-6)
Supplement: Supplementary file 4 — Reporting Summary [file 41467_2025_63118_MOESM4_ESM.pdf]

nature portfolio

Corresponding author(s): Simone Astori, Carmen Sandi

Last updated by author(s): Jul 22, 2025

Reporting Summary

Nature Portfolio wishes to improve the reproducibility of the work that we publish. This form provides structure for consistency and transparency in reporting. For further information on Nature Portfolio policies, see our Editorial Policies and the Editorial Policy Checklist.

Statistics

For all statistical analyses, confirm that the following items are present in the figure legend, table legend, main text, or Methods section.

n/a	Confirmed	
☐	☒	The exact sample size (n) for each experimental group/condition, given as a discrete number and unit of measurement
☐	☒	A statement on whether measurements were taken from distinct samples or whether the same sample was measured repeatedly
☐	☒	The statistical test(s) used AND whether they are one- or two-sided<br>*Only common tests should be described solely by name; describe more complex techniques in the Methods section.*
☒	☐	A description of all covariates tested
☐	☒	A description of any assumptions or corrections, such as tests of normality and adjustment for multiple comparisons
☐	☒	A full description of the statistical parameters including central tendency (e.g. means) or other basic estimates (e.g. regression coefficient) AND variation (e.g. standard deviation) or associated estimates of uncertainty (e.g. confidence intervals)
☐	☒	For null hypothesis testing, the test statistic (e.g. F, t, r) with confidence intervals, effect sizes, degrees of freedom and P value noted<br>*Give P values as exact values whenever suitable.*
☒	☐	For Bayesian analysis, information on the choice of priors and Markov chain Monte Carlo settings
☒	☐	For hierarchical and complex designs, identification of the appropriate level for tests and full reporting of outcomes
☐	☒	Estimates of effect sizes (e.g. Cohen's d, Pearson's r), indicating how they were calculated

Our web collection on statistics for biologists contains articles on many of the points above.

Software and code

Policy information about availability of computer code

Data collection	Confocal image acquisition: LAS-X v. 3.5.7.23225, Leica Microsystems.<br>Fiber photometry: Doric Lenses Neuroscience Studio v. 5.1.3.0, Doric Lenses Inc.<br>Electroencephalography: Sirenia Acquisition & Sleep Feedback Pro v. 2.2.2, Pinnacle Technology Inc.<br>Slice electrophysiology: pClamp v. 10.6, Molecular Devices, LLC<br>Slice imaging: ZEN 2012 v. 14.0.21, ZEISS Microscopy
Data analysis	Confocal image analysis: ImageJ Fiji v. 1.54f (https://imagej.net/software/fiji/); Qupath, v. 0.4.4 (https://qupath.github.io/); ABBA (https://abba-documentation.readthedocs.io/en/latest/)<br>Fiber photometry: Python version v. 3.11.3, Python Software Foundation<br>Electroencephalography: Sirenia Sleep Pro v. 1.7.6, , Pinnacle Technology Inc.; Python v. 3.11.3, Python Software Foundation<br>Slice electrophysiology: pClamp v. 10.6, Molecular Devices, LLC<br>Statistical analyses: Graphpad Prism v. 10.3.1, GraphPad Software<br>Custom codes are available here: https://doi.org/10.6084/m9.figshare.29195285

For manuscripts utilizing custom algorithms or software that are central to the research but not yet described in published literature, software must be made available to editors and reviewers. We strongly encourage code deposition in a community repository (e.g. GitHub). See the Nature Portfolio guidelines for submitting code & software for further information.

Data

Policy information about availability of data

All manuscripts must include a data availability statement. This statement should provide the following information, where applicable:

- Accession codes, unique identifiers, or web links for publicly available datasets
- A description of any restrictions on data availability
- For clinical datasets or third party data, please ensure that the statement adheres to our policy

No restrictions will be placed on data included in this study. All data supporting the findings of this study are available within the article and its supplementary information file, and raw data can be obtained from the corresponding author.

Research involving human participants, their data, or biological material

Policy information about studies with human participants or human data. See also policy information about sex, gender (identity/presentation), and sexual orientation and race, ethnicity and racism.

Reporting on sex and gender	N/A
Reporting on race, ethnicity, or other socially relevant groupings	N/A
Population characteristics	N/A
Recruitment	N/A
Ethics oversight	N/A

Note that full information on the approval of the study protocol must also be provided in the manuscript.

Field-specific reporting

Please select the one below that is the best fit for your research. If you are not sure, read the appropriate sections before making your selection.

☒ Life sciences ☐ Behavioural & social sciences ☐ Ecological, evolutionary & environmental sciences

For a reference copy of the document with all sections, see nature.com/documents/nr-reporting-summary-flat.pdf

Life sciences study design

All studies must disclose on these points even when the disclosure is negative.

Sample size	No statistical methods were used to predetermine sample size. Suitable sample size were estimated based on previous experience and are compatible with those used in the field, e.g.:<br>Thankachan, S. et al., Sci Rep 1–16 (2019), doi:10.1038/s41598-019-40398-9<br>Kjaerby, C. et al., Nat Neurosci 25, (2022), doi: 10.1038/s41593-022-01102-9<br>Fernandez, LM. et al., eLife (2018), doi: 10.7554/eLife.39111<br>The effect sizes are reported in Supplementary Table 1.
Data exclusions	For EEG experiments, 13 out of 59 animals were excluded based on poor or mistargeted viral expression, incorrect fiber placement or insufficient quality of EEG signal (high amount of noise and artifacts).<br>For patch-clamp experiments, cells that presented a change of >20% in series and input resistance across the recording were excluded. This accounted for ~15% of the recordings.
Replication	Experiments were repeated as indicated in detail for each figure panel and as described in the methods.
Randomization	Animals were assigned randomly to experimental groups to avoid litter, cage and batch effects.
Blinding	Given that the experimental design in the main datasets is focused on comparisons between baseline and stimulation conditions rather than between genotypes, blinding for group allocation was not implemented. However, analyses of RNAscope, fiber photometry, and EEG optogenetic experiments were conducted using custom-made scripts or automated detection methods, minimizing potential experimenter bias.

Reporting for specific materials, systems and methods

We require information from authors about some types of materials, experimental systems and methods used in many studies. Here, indicate whether each material, system or method listed is relevant to your study. If you are not sure if a list item applies to your research, read the appropriate section before selecting a response.

Materials & experimental systems

n/a	Involved in the study
☐	☒ Antibodies
☒	☐ Eukaryotic cell lines
☒	☐ Palaeontology and archaeology
☐	☒ Animals and other organisms
☒	☐ Clinical data
☒	☐ Dual use research of concern
☒	☐ Plants

Methods

n/a	Involved in the study
☒	☐ ChIP-seq
☒	☐ Flow cytometry
☒	☐ MRI-based neuroimaging

Antibodies

Antibodies used	rabbit anti-Chromogranin A (abcam, #ab15160; Lot GR3205971-6; 1:250)<br>mouse anti-parvalbumin (Synaptic Systems, #195011, Lot 1-13; 1:500)<br>donkey anti-rabbit IgG Alexa Fluor 647 (ThermoFisher Scientific, #A31573; 1:1000)<br>goat anti-mouse IgG Alexa Fluor 488 (ThermoFisher Scientific, #A11029, 1:1000)
Validation	anti-Chromogranin A:<br>https://www.abcam.com/en-us/products/primary-antibodies/chromogranin-a-antibody-ab15160<br>A negative control for anti-Chromogranin A was performed and included in the supplementary data.<br>anti-parvalbumin:<br>https://www.sysy.com/product/195011

Animals and other research organisms

Policy information about studies involving animals; ARRIVE guidelines recommended for reporting animal research, and Sex and Gender in Research

Laboratory animals	This study was conducted on transgenic mouse lines with C57BL/6J background:<br>B6(Cg)-Crhtm1(cre)Zjh/J (The Jackson Laboratory, Strain #:012704 RRID:IMSR_JAX:012704)<br>B6.Cg-Gt(ROSA)26Sortm27.1(CAG-COP4*H134R/tdTomato)Hze/J (The Jackson Laboratory, Strain #:012567, RRID:IMSR_JAX:012567)<br>B6.FVB(Cg)-Tg(Ntsr1-cre)GN220Gsat/Mmucd mice (Ntsr1-Cre, The Jackson Laboratory, RRID:MGI:4358487)<br>CRH-IRES-Cre x Ai27D mice and Ntsr1-Cre x Ai27D mice were generated in house by crossing the mouse lines above.<br>Mice were aged between postnatal days P28-84.
Wild animals	No wild animals were used in this study.
Reporting on sex	For ex-vivo experiments, both male and female mice were included. Data are reported segregated by sex in Fig 1 and Suppl. Fig. 7 and for all datasets in the Source Data file.<br>For EEG recordings, only male mice were included to avoid potential confounding factors derived from the impact of sex hormones on sleep architecture (Swift, KM. et al., Front. Neurosci. (2024), doi.org/10.3389/fnins.2024.1426189).
Field-collected samples	No field-collected samples were used in this study.
Ethics oversight	Animal care and experimental procedures were carried out in accordance with the Swiss Federal Guidelines for Animal Experimentation and were approved by the Cantonal Veterinary Office Committee for Animal Experimentation (Vaud, Switzerland).

Note that full information on the approval of the study protocol must also be provided in the manuscript.

Plants

Seed stocks	N/A
Novel plant genotypes	N/A
Authentication	N/A
